# "The Hospital" Nursing Mirror

**Published:** 1898-12-24

**Authors:** 


### "The Hospital" Nursing Mirror.

Being the Nursing Section of "The Hospital."

[Contributions for this Section of "The Hospital" should be addressed to the Editor, The Hospital, 28 & 29, Southampton Street, Strand, London, W.C., and should have the word "Nursing" plainly written in left-hand top corner of the envelope.]

### News from the Nursing World.

### THE PRINCESS CHRISTIAN ON NURSES.

All our Royal family show a keen interest in the allied arts of medicine and nursing, and the Princess Christian takes a very active part in promoting the interests of our nurses. She sympathises with the feeling born of actual experience with the arduous nature of a true nurse's work. Thus, in writing on "Nurses and their Vocation" upon what "being a nurse" means, it is with a full knowledge of the import of her words that she answers: "Years of hard work, of education of mind and body, of experience combined with sympathy, and of complete self-abnegation. Nursing is the highest, noblest duty which a woman can fulfil, provided she does so rightly, earnestly, and with singleness of purpose."

### PRINCESS BEATRICE AT ROMSEY.

H.R.H. the Princess Henry of Battenberg proposes sometime soon, probably in the second week of the New Year, to visit Romsey for the purpose of opening the Jubilee Nurses' Institute.

### OUR CLOTHING DISTRIBUTION.

It is with much gratitude and pleasure that we acknowledge the kindly and liberal gifts of our readers to our Clothing Distribution Fund. They are more numerous than last year, and some postal orders enclosed shall be expended to the greatest advantage. The following ladies have sent parcels: Nurse K. Prior, Nurse Lizzie (Edinburgh), Miss Lucmyer, Nurse Groom (socks and P.O.O., 2s.), "Peggie," Nurse Clarke, M. G. Thring, Miss Henslowe, Miss Kate Laczkovic, and Miss Alice M. Newson (P.O.O., 5s.).

### OUR CONVALESCENT FUND.

We wish to bring strongly before our readers the claims of "The Hospital Convalescent Fund." This fund is chiefly maintained by the gift of nurses in small sums of about 2s. 6d. and upwards; and by it convalescent nurses, who need just the rest and change that fits them to enter anew the battle of life, are enabled to enter some convalescent home in a locality medically recommended to each particular case to receive it. The average holiday the Fund provides is three weeks, the lowest cost 37s. 6d.—surely a small sum for so great a boon—whilst when the need is imperative a few shillings more are given. If one remembers that it takes eight half-crowns to make £1, it is easy to calculate how many subscriptions are needed to send one patient for a holiday. Although we seldom ask for money—it has rarely been needed so far—the applications are now so frequent that the funds are running low. Will our readers remember their sick sisters, and send contributions to the Hon. Secretary at this office?

### SIOUX INDIAN NURSES.

Nursing is the heritage of no one nation, and that there are now in the Third Division Hospital, Jacksonville, Florida, four nursing sisters direct descendants of some of the Sioux tribes is a romantic illustration of the fact. It is no secret that the redskin damsels possess many of the qualities of a good nurse, and by constitution and temperament will doubtless be able to work under conditions impossible to the white woman. It also appears that these American Indian women are banded together in a sort of guild or order, which admits none but Indian girls. Quite recently its offer of nurses for the front has been accepted by the United States authorities.

### "TRUTH'S" DOLL SHOW.

"Truth's" Doll Show at the Albert Hall, which has been held this week, has become an annual institution, and the source of an immense amount of pleasure. The children in our hospitals look forward to the packages which make Christmas Day and those coming after it bright with dolls and toys. The donors and competitors who have lavished such a wealth of ingenuity and kindly feeling can in nowise be without their reward, even if they are not the lucky winners of one of the tempting prizes also on view; whilst the numbers that visit the show, big folk as well as small, spend a very delightful hour in seeing how amply this agency caters for the joy of the poor sick little ones. This year funds have been badly needed, but the eagerly-awaited toys are nevertheless ready for their future owners.

### THE DOMINICAN SISTERS IN RHODESIA.

The action of the townsfolk in Bulawayo in calling a public meeting to appoint a representative committee to consider some scheme as to the best manner of showing the public appreciation in a permanent form of the work done by the Dominican Sisters in nursing the sick in the hospitals of Rhodesia during the last six years is appropriate, and the unanimous vote of thanks and gratitude to the Sisters for their services to the community must have caused them great satisfaction and pleasure, especially as they have relinquished work in the hospitals, and will in future devote themselves to missionary enterprise. Four of the Sisters were this year awarded the Royal Red Cross for their services.

### THE LONDON MEDICAL MISSION.

The London Medical Mission, by which so much good is done in the slums of St. Giles, has issued an occasional paper in the interests of the work. Money is needed for the following five branches: (1) The general fund; (2) the Lambeth sick and poor fund; (3) the convalescent home fund; (4) the kitchen and special fund; and (5) for the holiday house fund. The address is 33 and 34, Short's Gardens, Endell Street, St. Giles, London, W.C.

### DOMESTIC ECONOMY AT EDINBURGH.

The annual balance-sheet of the Edinburgh School of Cookery and Domestic Economy (Limited) proves one very satisfactory thing, namely, that technical schools are dividend-paying concerns if rightly managed, in addition to the advantages offered in the shape of domestic welfare. Germany has long had schools of housewifery, and a year's training at one of them is regarded as essential to a girl's education—a happy state of affairs that we as a nation would do well to imitate. The trend of public opinion is certainly in that direction, as is witnessed by the increased usefulness of the Edinburgh school.

### THE SCHOOL OF BIBLE STUDY.

This is the title chosen to describe a series of lectures to be delivered at various centres of the metropolis by Professor W. W. White, of Yale University, U.S.A. Professor White was late professor of Hebrew and Old Testament Literature at the Xenia Theological Seminary there, and the aim set forth by the committee in the proposed series is a comprehensive study of the Bible as a whole, and the relative bearing of one portion on another. The president is Lord Kinnaird, and the invitation to undertake this work was given to Professor White by the Young Men's Christian Association, the British College Christian Union, and the Young Women's Christian Association. Full particulars may be obtained from Mr. L. Meager, 26, George Street, Hanover Square.

### YORK NURSES.

News of sales of work grows plentiful as the end of the year approaches. Nurses and those interested in them make renewed efforts to banish winter's gloom by Christmas cheer and festivities, and the sales are usually to provide necessities and comforts for the sick and those who tend them. The Nursing Home at York has been open now for nearly twenty-eight years, and a two days' sale is an annual event for providing funds for the poor. This year a number of ladies, in token of appreciation for the nurses' work, have embroidered a white rose on a black ground, with the initials, "Y. N. H.," underneath, in form of a badge, which will for the future be worn by the nurses attached to the staff. The Dean of York was present on the opening day of the sale, and presented some nine-and-twenty of these badges, reading the recipients a little homily on its signification (purity of heart), the privileges of their calling, and their responsibility in maintaining the present high reputation of the Home to which they were attached.

### AN APPEAL.

The Farringdon General Dispensary and Lying-in Charity, which since 1828 has done useful work amongst the sick and destitute, especially in helping poor mothers, is languishing for want of money. Most of the firms in this neighbourhood have been turned into limited liability companies, which having, as someone remarks, "neither bodies to be kicked nor souls to be saved," have cut short their contributions to charities. Thus it happens that, unless £100 can be collected to pay off the existing debt, and a sufficient income maintained, the institution must be closed, as the committee are without funded property. The number of patients annually attending is about 20,000. The hon. secretary, Mr. W. K. Taunton, 17, Bartlett's Buildings, will acknowledge any subscriptions and give any information required.

### A SECOND TERM PLAGUE NURSING.

Our attention has been drawn by a correspondent to the fact that whilst the nurses on plague duty have been asked to sign an agreement to serve for a second year no increase of salary has been offered them. This could well have been done, and no doubt such a course would have induced a larger number of nurses to remain. Moreover, it would only have been fair, for this would cause much less expense to the authorities engaging them than would be occasioned by sending home for substitutes, travelling expenses and allowances forming of necessity heavy items in the expenditure of plague nursing, to say nothing of the fact that those who have spent a year in India are not only more or less acclimatised, but are more valuable public servants, for the newly-imported nurse is always at a disadvantage for the first few months.

### PLAGUE TROUBLES AGAIN.

The terrible uncertainty that hovers over plague-infected India as to when and where the justly-dreaded epidemic may next make its appearance is truly awful, and we can fully sympathise with the desire of the residents of Ooty, Ootacamund, Madras, in their anxiety to have the migration from the plague-infected districts of Bangalore under the inspection of a lady nurse versed in the work. After the terrible object lessons of the past two years it is difficult to understand how it is possible for them to let any consideration whatever stand in the way of securing such assistance, and the latest news—of course a month old—that a question of salary and the difficulty of obtaining a nurse was causing the loss of precious time and opportunity is much to be regretted.

### SHORT ITEMS.

On Tuesday last week Mr. Frederick F. Slocombe gave his second magic lantern entertainment to the in-patients and nurses at the Royal Orthopædic Hospital, W. About 200 slides were exhibited, some accompanied by popular songs of the moment.—Misses Gladys and Hilda Willoughby, of Edale House, Newark, took the trouble to arrange a sale of work on behalf of the funds of the District Nursing Association, which brought in £20.—Dr. Arkle (Charing Cross Hospital) was particularly pleased with the examination of third-year nurses just held at the Lewisham Infirmary. The successful candidates were F. K. Whitmarsh, A. C. Bartlett, M. Morpeth, and N. Weddall (equal); S. Burton, J. C. Marr, M. Sheridan, and R. E. Clarke.—Germany is following an English precedent, and is about to establish a college for training nursemaids at Brussels. Lectures will be given in physiology, diet, pathology, and hygiene, and the course will be capped by examinations and a diploma.—Miss Bermingham, who has just passed her final examinations in the General Hospital, Toronto, Canada, has been appointed superintendent of nurses of the Hospital at Lebanon, Kentucky, U.S.A.—Thanks to one or two windfalls in the shape of donations, and the result of entertainments, the committee of the "Gilbert Adams" Nursing Institution for the parish of Beaminster have been able to furnish the nurses' rooms to a certain extent and to end the year with a small balance in hand. Nurse Thomson has replaced Nurse Proffitt, who left last spring for Australia.

### Nursing in Diseases of the Throat, Nose, and Ear.

By Macleod Yearsley, F.R.C.S.Eng., Assistant Surgeon to the Royal Ear Hospital, Surgeon-in-Charge of the Department for Diseases of the Throat, Nose, and Ear, the Farringdon General Dispensary, Hon. Surgeon for Diseases of the Throat and Ear, the Governesses' Home, &c.

### X.—TRACHEOTOMY AND ITS AFTER-TREATMENT.

Tracheotomy is one of the emergency operations of surgery, and the nurse should therefore know not only something about it and its indications but she should possess as good a knowledge as she can obtain of its after treatment. Owing to its importance a special one of this series of articles has been devoted to it. Tracheotomy is an operation which may be to the surgeon the easiest or the most difficult. In an adult, quiet under good anæsthesia, it is as plain, straightforward, and easy as any operator could wish, but in a child, fat and short of neck, with the larynx jumping up and down and the veins enlarged by impending asphyxia, it may be one of the most difficult of operations, especially when done at night and with bad illumination.

### The Indications for Tracheotomy.

Tracheotomy may be rendered necessary whenever death threatens from laryngeal obstruction, which is not at once amenable to other treatment. It may, therefore, be required in the following conditions: (1) Diphtheria; (2) syphilitic and tuberculous ulceration; (3) malignant disease of the larynx; (4) acute laryngitis (especially in œdema of the glottis); (5) certain spasmodic affections, *e.g.*, tetanus, aneurism of the thoracic aorta; (6) foreign bodies in the air passages; (7) as a preliminary to other operations. The preparation of the patient, when such is possible, does not differ from that for other operations; as a rule, tracheotomy being done for an emergency, no preparation, or a very hurried one only, is possible.

### The Operation.

Tracheotomy means the opening of the trachea, or windpipe, in order to form a new passage (which may be temporary or may have to be permanent) for the patient to breathe through and the insertion of a tube into the trachea to keep that opening patent. There are two forms of the operation, the "high" and the "low." In the former the trachea is opened above, in the latter below the isthmus of the thyroid gland, which crosses it opposite the second and third rings. The "low" operation is the more difficult, because the trachea lies more deeply. The instruments required (and which the nurse may have to put out) are as follows: Scalpel, dissecting forceps, Spencer Wells' forceps (several pairs), blunt hooks, sharp hook, director, ligatures, sutures, needles, scissors, tracheotomy tubes, red-rubber catheter and syringe attached (to suck any fluid out of the trachea), absorbent wool swabs for sponging. Antiseptics and hot water as usual. Should the nurse have to arrange the operating table she should see that a small, narrow pillow is at hand to place beneath the patient's neck.

Of the operation itself it would be out of place to speak here, but a useful caution is necessary when the operation is performed for diphtheria, namely, both operator and nurses should always stand aside at the moment the trachea is opened; its contents may be ejected with much force, and diphtheritic infection may thus be incurred by direct contact.

It is advisable that the nurse should learn something regarding the tracheotomy tube and its different forms. Tracheotomy tubes consist of an outer tube attached to a shield and an inner tube. The best tubes are those of Durham, Bryant, and Parker. The nurse should see these tubes and become conversant with the special features of each. Durham's tube possesses an inner canula, which is jointed lobster tail fashion, and is provided further with a lobster-tail "pilot" on a handle. Parker's tube is angular, and much to be preferred on anatomical grounds; those tubes which are curved rather than angled are liable to impinge with their lower extremity on the anterior wall of the trachea, thus tending to produce ulceration there; such a bad-shaped tube is the one known as Fuller's. At either side of the shield are slits for tapes; these tapes, which must be long enough to tie comfortably round the patient's neck without producing any constriction, should be ready threaded on the tube before the operation.

### After-treatment.

The future of a tracheotomy case depends very largely upon careful nursing, and the nurse who is put on such duty must keep continually before her mind the great responsibility committed to her charge. It is best that there should be two nurses to relieve each other, for the patient cannot, without danger, be left for a moment alone. Expectoration must be encouraged, and to some extent this is done by keeping the room atmosphere moist. Sudden obstruction of the tracheotomy tube is most often due to inspissated mucus, not to the diphtheritic membrane; this thick mucus is secreted usually about twenty-four hours after the operation, and after three or four days the discharge becomes thinner and more puriform. In order to keep the patient in a warm moist atmosphere most surgeons prefer the tent, but some operators are of opinion that the unvarying rule of cottenting and the use of steam is disadvantageous. It must be remembered that children suffering with membranous laryngitis are in a weakly condition, and consequent semi-complete seclusion with so little admission of air tends to increase the asthenia and sepsis. Some, therefore, prefer to have the patient in a bed screened from draughts, using steam if needful. A tent, when required, can be readily improvised by fastening four vertical pieces of wood to the corners of a cot, connecting them by four horizontal pieces, and throwing a sheet over all. One side of the cot is left partly uncovered to allow of the entrance of steam from a steam kettle. Large clothes-horses can also be used round the bed. A uniform temperature of 65 deg. or 70 deg. Fahr. should be preserved, and all draughts carefully avoided. The nurse who has charge of the case should lay a double fold of fine gauze, wrung out of warm water, over the mouth of the tracheotomy tube; this is to filter the air, and requires careful and constant watching in order that any clots, mucus, or membrane when ejected may be at once dexterously brushed away. At intervals of about every twenty minutes the nurse should remove the inner tube and cleanse it; should it be coughed out during the intervals it should be carefully cleansed before it is reinserted. The cleaning may be done with warm carbolic lotion, but if the secretions dry and cling to it, it will be found that a warm solution of bicarbonate of soda (gr. v. to xx. to ℥i.) will be the best to use. The lumen of the tube is generally cleaned with a feather—why a feather is the correct implement one cannot say, probably it is a mere custom—and the feathers used for the purpose should be ordinary poultry wing feathers, carefully washed in carbolic, and with all loose barbs removed, a precaution particularly necessary when the feather is to be used for cleaning the tube *in situ*. With regard to these directions as to cleansing, &c., I would impress upon the nurse that such manipulations are very easily overdone. A nurse should endeavour, when engaged in the care of such cases, to be guided by her common-sense. To sit by the side of a child and stuff feathers down its throat at intervals of from half to two minutes, varying the performance by extracting, cleansing, and reinserting the inner tube every ten minutes, and finally dropping off to sleep from the exhaustion consequent upon such unceasing labours, is not the common-sense method of nursing a tracheotomy case. If the nurse will remember that she has to prevent the tube from becoming obstructed, that she must therefore exercise unceasing vigilance without exhausting the patient by meddlesome interference, that she must on no account allow herself to fall asleep, and that she must never, upon any pretext or under any consideration, remove the outer tube, she will be more than half way towards competency to take charge of a case of tracheotomy. Another very important item is the feeding of the patient. Every time he wakes he must be fed. Often there is much difficulty in getting sufficient food taken on account of the pain in swallowing, the impairment thereof from the presence of the tube, &c., and the nurse will have to devote much time and patience to coaxing the child to take plenty of nourishment. Brandy and beef tea may be given freely. Occasionally it is better to feed the patient at regular intervals with stated amounts by means of an œsophageal tube; this, however, will of course be seen to by the surgeon himself. At the end of 24 hours it is usual to change the outer tube. This is done by the surgeon, and the nurse should see that the new tube which is placed ready for him has a guide with it. Lastly, the nurse must see that, in all cases of diphtheria, every article that quits the room is thoroughly and completely disinfected. All swabs, folds of gauze, feathers, &c., that are used for the tracheotomy tubes should be at once destroyed by burning, and last, but often most difficult task of all, great vigilance must be exercised over the attentions of relatives and friends of the patient.

### Lectures on Medical Relief.

Under the Auspices of the Charity Organisation Society.

### SICK ASSURANCE.

The lecture recently given at the Portman Rooms by Mr. F. G. P. Neison, F.S.S., F.I.A., was on the subject of "Sick Assurance."

Mr. Neison began his remarks by a brief review of the growth and history of friendly societies in this country. Coming to the present time, Mr. Neison explained the general principles upon which friendly societes were based, and the technical meaning of the word "sickness," which in connection with "sick assurance" was not meant in its strict medical sense, but as implying "disqualification for labour." Thus so slight an ailment as a pricked finger, from its natuie interfering with the wage-earning capacity of a tailor, but a matter of no importance to, say, a messenger, would qualify the former for "sick pay," but not the latter. It was in consequence of the difficulty in differentiating between the comparative disabilities in various forms of illness and in various trades that the rule that members of friendly societies must be wage-earners had become a fixed one, and this rule had been the chief obstruction so far to the admission of women and children to the benefits of sick assurance. School children were now eligible, however, and the Oddfellows and Foresters now admitted women.

Mr. Neison then went on to explain the plans adopted to circumvent malingering, and the graduated scale of pay given in cases of long-continued illness, afterwards dealing with "incidence of sickness risk," and giving the results of inquiries made into this subject. Speaking of the comparative death and sickness rates in various trades and orders, the lecturer noted the fact that agricultural labourers were remarkable for a low rate of mortality but a high rate of sickness, and in connection with the total abstinence question he mentioned that while the Independent Order of Rechabites were noticeable for a low death-rate, their principle of total abstinence had, according to the last returns, shown no difference in the sickness rate. Mr. Neison caused some amusement by his frank avowal that he attached no importance whatever to medical certificates as reliable testimony in cases of illness and by his denunciation of "clergy and women" as upholders of the "financially rotten system of slate clubs" and similar institutions. He strongly urged the advisability of joining the big societies, such as the Oddfellows or the Foresters, &c., declaring his conviction that the day had gone by for county orders and that the friendly society of the future was the affiliated order.

With regard to suggested reforms in the future, there was the proposal that membership of friendly societies should cease at the age of 65, a pension allowance then being made in lieu of further sick pay. But here came in the difficulty, that it was almost impossible to persuade the working man that he would live to reach the age of 65, though statistics proved that, taking any adult meeting of people, fully one-half of them would reach that age. And here Mr. Neison gave an interesting account of the extraordinary difficulty experienced in the case of a certain railway company when it was wished to encourage the employés to enter upon such an arrangement—purely in the interest of the men as it was—because of their firmly-rooted conviction that they would not live to enjoy the advantages offered, and in spite of the fact that the money paid in by them was returnable if death took place before the age at which the pension became due. Then there was the suggestion of Government subsidies, a principle to which the speaker himself was entirely opposed; the system by which a man might become his own landlord, providing for his own sickness and for his wife and family should he die early; there was also the assurance of apprenticeship for his sons, a system now much gone out; and, lastly, the advantages offered by the savings bank. Mr. Neison concluded a lecture, which had been listened to with much interest, by pointing out that a magnificent exhibition of thrift had been shown by the friendly societies during the last fifty years.

After the lecture several questions were raised by some of the audience, one member of the Independent Order of Rechabites controverting Mr. Neison's remarks concerning the non-influence of total abstinence on the sickness rate. One lady asked about assurance for women, and, in reply, Mr. Neison again emphasised the difficulties in the way of sickness benefits for women, unless they were wage-earners. The Order of Foresters now numbers some six thousand women among its members, and that of the Oddfellows some four thousand.

### Presentations.

Tiverton friends took advantage of the marriage of Miss O'Reilly, formerly matron of the infirmary of that place, to present her with silver tea-spoons, sugar spoon and tongs, and a pair of silver salt cellars and spoons.

On December 19th, the occasion of the nurses' annual dance, Miss Jessie Murray, matron of the Huddersfield Nurses' Home, was presented with a handsome gold curb bracelet by past and present members of her staff.

### Life in a Cottage Hospital.

By a Nurse-Matron.

Many nurses have an idea that the life of a cottage hospital matron must necessarily be dull and monotonous; and, fresh from the varied experience of a large training school or the rush and excitement of an accident hospital, one is apt at first sight to take their view of the matter. After some years' experience of this branch of nursing, I look back with no little amusement to the feelings of utter despair which filled me during the first two or three months I spent in charge of a cottage hospital. The few cases then in the wards seemed to me of a most uninteresting character. I was new to the management of a kitchen, and of a buxom country servant so much bigger than myself that I felt somewhat intimidated in her presence; worst of all, there were sometimes so many spare hours that time hung heavy on my hands, and I felt strongly inclined to run away in spite of the kindness shown me by all the people with whom I came into contact. However, the stage of *ennui* was soon over, and I awoke to the fact, of which I am daily more convinced, that nowhere in the nursing world is there more scope for the exercise of a nurse's best faculties, of her tact, patience, and resource, than in a cottage hospital; added to which, having perhaps less than a dozen patients in at a time, and having them often in one's sole charge, it is possible to take a friendly interest in the life of each one, and to obtain an influence that need never be lost, for in agricultural districts, at any rate, people are for the most part stationary, and seldom leave their village homes for pastures new. It is no unusual thing to have in the course of two or three years various members of the same family as patients, and it is very gratifying to hear such a speech as "Tommy doesn't mind coming into hospital a bit, Sister, 'cause Rosie was so happy here"; the old patients, too, will come to confide their troubles and difficulties, and to ask for sympathy and often for advice. My lot as matron has been a happy one, owing, in great part, to the unfailing kindness and hearty co-operation of the medical staff, who take a great interest in the welfare of our little hospital. It has always been our ambition not only to give all the physical benefit possible, but to aim at sending out each one a little better both morally and mentally, and to instil into their minds the elementary principles of sanitation as applied to their houses. We find that most of our patients have an ineradicable and apparently instinctive dislike to ventilation and water, especially water, their ablutions, if not carefully superintended, being most superficial. I remember one old man who when he saw the hot bath prepared for him on admission, wept, and said he had never had a bath since he was a baby, and he was sure it would be "the death of him." We had less sympathy with a young man of a rather better class who, when confronted with the "ordeal by water" said, "Madam, I must respectfully but firmly decline. I am twenty-seven years old, have never had a bath, and—" Nurse interrupted him by remarking that it was high time he had one, and to his intense disgust he was helped into the water and "respectfully but firmly" bathed by the porter. Not long ago I went to see a favourite old woman, and found her suffering from a severe cold. "Why, granny," I said, "how did you get this dreadful cold?" "Well, M'm," was the reply, "I don't rightly know, but I can only think 'twas because I washed my feet!" Perhaps even better was the saying of an old lady admitted into hospital the other day. In a mild and tentative fashion I hinted at the bath in store. "Indeed, my dear, I dare not," she exclaimed, "for if ever I even wash myself I am sure to take cold."

All who work amongst the poor must find the deeply-rooted prejudices of ignorance harder to combat than any other form of opposition. In East Anglia superstition still lingers, and occasionally, in return for attentions which have pleased them, and by way of showing their gratitude, our old female patients will give us valuable "wrinkles." One "wise woman" assured me that the only infallible remedy for whooping-cough was to tie the sufferer up in a sack and drag him or her through a bramble bush four times! The treatment to be repeated every morning until the cough disappeared. Many people, she added, considered fried mice a specific for the same disease, but personal experience led her to believe in the superior potency of the bramble bush. Another old dame once told me that her family cure for a gathered finger was to apply a poultice of smashed snails (shells included). I left the ward hurriedly to avoid further details.

Among our patients we have a small percentage of infants usually suffering from malnutrition. The routine diet of the agricultural labourer's baby is what is popularly known as "bread mess," that is to say, bread soaked in water, with a little sugar added to make it palatable. It is nothing unusual for an infant to take one or two mugs of this food daily from its birth; the fittest not only survive, but appear to thrive on the rather heavy diet; but one wonders if their digestion is not being ruined for after life. Those who are not able to digest it either die or arrive at the hospital in the arms of a weeping mother, who informs one that she can't think why baby does not get on. Of course, in a village fortunate enough to possess a trained district nurse, much of this suffering could be prevented, but in a really poor district a nurse is, alas! an unattainable luxury.

In winter time women often come with a note from one of the medical officers, "Kindly show bearer how to make a poultice," and in this connection I remember a very funny incident told me by a doctor. A poultice was ordered for a cottage patient, and the neighbour who was acting as nurse was shown how to make it. At the doctor's next visit, he asked the patient if the poultice had not proved warm and comforting? He was surprised when she said, "No." But his wonder vanished when he found the poultice applied, under the nightgown, but over a thick vest and chemise.

One of the great difficulties of cottage hospital nursing is that in many instances the hospital income is so limited that a night nurse cannot be provided, and the matron is liable to be disturbed night after night, and even to be on duty thirty-six or forty eight hours at a stretch. Still, given good health, enthusiasm, and a love of her profession, a nurse can find compensation in the gratitude of her patients, and the knowledge that her trained hands are saving them needless suffering.

### Marriage.

On December 17th the marriage of Mr. J. Malcolm, clerk to the Guardians of the Easington Union, Durham, and of Nurse Annie Cooper, late matron of the Fever Hospital, Easington, was celebrated at St. Nicholas Church, Durham. The noteworthy feature of the ceremony was the costume of the bride and her maids, who wore their nurse's uniform.

### "The Hospital" Convalescent Fund.

With much pleasure we acknowledge a contribution of 2s. 6d. from Nurse E. Jarman.

### Christmas Books.

"King Alfred's Viking"* is a capital book for boys. It is full of interest and adventure, and suited to stir the brain of the least adventurous of boys. The general details and course of events given in the story are, as far as regards the private life and doings of King Alfred, from his life as written by his chaplain, Arser. That Alfred put his first fleet into the charge of certain Vikings is well known, though Mr. Whistler tells us in his preface that the name of their chief has never been given. The hero of the story is this adventurous chief himself, and there are good historic grounds for the position given to him in the book, as leader of the newly-formed fleet. Mr. Whistler tells his tale in the first person, and it is King Alfred's Viking who is the narrator, a man whose early childhood was associated with the glare of a burning hall, with the shouts of savage warriors, among whom he, a ten-year-old boy, was brought up, but his later career, as here described, is hardly less thrilling in its details than are the faint recollections of those earlier times. The book is one all boys will love.

Of a less exciting nature is "The Dormitory Flag."† This is a story of schoolboy life at Barnbury College, and a certain ring of truth about the narrative leads one to surmise that the plot, as the characters also, are taken from real life. There are eight fine illustrations by W. H. Margetson. "A Fighter in Green,"‡ by Herbert Hayens, is a tale of Algeria, the plot of which, and it is one of warfare, runs through twenty-seven chapters, but the interest never flags, and is well maintained throughout. "French and English."§ The scene of this book is laid in Canada, and is the story of the storming of Quebec. Wolfe figures, of course, throughout the narrative, firstly as the soldier at home, and in the subsequent dramatic episodes of his all too brief career. This story, besides providing romantic reading, has a historical value of its own.

Quite a plethora of cookery books have been published this season and are before us for review, from among which we select "The Century Invalid Cookery Book,"|| written by Mary A. Boland and edited by Mrs. Humphrey ("Madge," of *Truth*). This would form an excellent Christmas gift to nurses, and be an acquisition to any household. The book contains explanatory lessons on the properties and value of different kinds of food, and recipes for the making of various dishes. Of equal value, though in a more limited compass, is "Cookery for Common Ailments."¶ The book is intended to serve as a practical dietetic guide to the invalid. At the end of the volume there will be found a number of recipes for the preparation of foods suitable to illness in its more acute stages, from which selection may be made with the sanction of the medical attendant. Then there is "A Year's Cookery,"** by Phyllis Browne, an expert on this all-important question of the table, and here we find dishes given for breakfast, luncheon, and dinner for every day of the year, with instructions for their preparations and their purchase.

* "King Alfred's Viking." By Charles W. Whistler. 2s. 6d. (London: Thomas Nelson and Sons, 1899).

† "The Dormitory Flag." By Harold Avery. 5s. (London: Nelson and Sons. 1899.)

‡ "A Fighter in Green." By Herbert Hayens. 5s. (London: Nelson and Sons. 1899.)

§ "French and English." By Everett Green. 5s. (London; Nelson and Sons. 1899.)

|| "The Century Invalid Cookery Book." 3s. 6d. (London: Fisher Unwin. 1898.)

¶ "Cookery for Common Ailments." By a Fellow of the Royal College of Physicians and Phyllis Browne. Limp cloth, 1s. (London: Cassell and Co. 1898.)

** "A Year's Cookery." By Phyllis Browne. Limp cloth, 1s. London: Cassell and Co., 1898.)

### Where to Go.

The New Gallery.—The winter exhibition this season is devoted to works by Sir Edward Burne-Jones. The gallery, which is in Regent Street, opens on December 29th.

### Miss Burns's Marriage.

### CONGRATULATIONS FROM NURSES OF THE PENSION FUND.

We publish below the names of the nurses who have already joined in the scheme to congratulate Miss Burns, daughter of the late chairman of the Royal National Pension Fund for Nurses, upon her forthcoming marriage. The names of all contributors will accompany the illuminated address, which will be on show when completed. The subscription is limited to sixpence, so that all may take part without taxing their resources, and contributions should be sent, with name and address, to the Editor, The Lodge, Porchester Square, W., as soon as possible. J. E. Roberts, S. A. Mallinson, Phillis Legate, Green, Barnes, Shoesmith, Rosa Smith, J. R. Dinwoodie, No. 580, E. M. Pearce, M. Buckworth, M. Donelly, E. Sweeting, L. Wilkinson, H. M. Coleman, M. I. Kyte, L. H. S. Blaydes, E. A. Barnes, F. Stockwell, E. Jones, No. 37, S. Dutton, Policy 2,336, A Policyholder, E. G. Goss, M. S. Weston, A. Domnall, J. Freese, T. Keogh, A. E. Smith, K. E. Richards, O. A. Richards, M. A. Barnes Groom, E. P. Machin, Anne Cole, A. O. Dickinson, M. A. Adkins, B. L. A. Thomson, M. Bellamy, J. A. Sealey, Jane McCosh Smith, S. J. Bellamy, J. G. Musgrave, S. Pinington, M. E. Barber, H. Tudor, S. A. Grigg, E. Freeborn, M. E. Palmer, Gowan, I. Spragge, J. Connell, Harrison, A. E. Smith, C. Tomlinson, A. Bridle, E. G. Savigny, H. Kent, A. Moore, C. E. J. Chaffer, H. I. Proschwitzky, Norman, A. Barker, M. A. Edwards, C. Cowen, F. L. Chapman, F. I. Claringbold, Policy No. 1,330, M. Prince, M. E. Davies, S. S. Elliott, E. Smith, M. J. Packwood, H. J. Jones, F. Shaw, M. Bristowe, Helen Booth, A. H. G. Macvicar, M. J. Love, J. Gerard, A. Pumphrey, P. B. Bloomer, M. G. A. Warner, S. A. Goodchild, M. Coverdale, Goold, J. E. Mount, L. C. F., E. Jeffrey, E. A. Fenton, Alice, M. L. Town, Hadly, S. A. Booth, E Butler, A. B. Woodward, Fanny Storm, J. E. Coombs, L. Harris, De la Chaumette, S. C. Borgaes, Maunder, C. E. Parker, L. F. White, J. Biddock, E. Keen Willcox, S. A. Carvosso, E. L. Brown, E. H. Gibson, Farrar, M G. Thring, C. J. Fenton, J. Cox, K. Cox, S. Talbot, E. Overton, A. M. Chaloner, A. McMillan, A. Megarry, E. Nicholson, A. Godwin, M. A. Soper, C. Miskin, C. M. Wreford, A. Peck, Ada, S. A. Clark, S. Lambert, Clarke, Turner, M. A. Harris, E. Clark, Lovell.

### Appointments.

The Military Infectious Hospital, Aldershot.—Miss A. E. Constance, who was trained at Gloucester General Infirmary, has been appointed Head Nurse here. She has previously been charge nurse at the Willesden Isolation Hospital, at the City Isolation Hospital, Bagthorpe, and at the South-Eastern Fever Hospital (Metropolitan Asylums Board).

Royal Hospital for Diseases of the Chest, City Road, E.—On the 19th inst. Miss Emily Kate Lynge was appointed Matron here. She was trained at King's College Hospital, and was afterwards sister of the same hospital for six years.

### Wants and Workers.

[The attention of correspondents is directed to the fact that "Helps in Sickness and to Health" (Scientific Press, 28 & 29, Southampton Street, Strand, London, W.C.) will enable them promptly to find the most suitable accommodation for difficult or special cases.]

Home for Crippled Lad.—"Matron" would be much obliged if anyone could give her particulars of a home in Scotland (not Quarier's) where a bright intelligent crippled lad could be received and educated free, as his parents are poor. He is seven years old and lost his legs through a carriage accident.

### Training in the Provinces.

(*Continued from page* 104.)

### SALISBURY GENERAL INFIRMARY (118 Beds).

### Terms of Training.

PROBATIONERS are received for a course of two years' training at the Salisbury General Infirmary, the age considered desirable being between 21 and 34. No salary is given to probationers, who pay a premium of £10 10s. for the two years and are required to provide their own uniform. The salaries of ward sisters begin at £25 per annum, rising to £30, and the hospital gives them indoor uniform. Three courses of lectures are given in the year, classes are held, and certificates granted after satisfactorily completing training. General fitness and two years' training are the essential qualifications for promotion to the charge of wards. Night duty is taken by probationers in their second year for periods of two months at a time.

A unique custom which prevails at the Salisbury General Infirmary is the admission of a certain number of "nursing students"—ladies who work in the wards for seven hours daily, attending for six months or a year, receive "certificates" from the committee if considered efficient, and provide their own board and lodging outside the hospital. They pay a premium of £5 5s., and are required to provide themselves with dresses "suitable in colour and material to their occupation." Candidates must be proposed and seconded by members of the committee of management; there is no limit in age for these "nursing students."

### Hours On and Off Duty.

The hours on duty for the day staff are between 7 a.m. and 9 p.m. During these hours two are allowed for recreation, and an hour and a half for meals. "Church time" is allowed every Sunday, and four hours off duty are given every second Sunday. Nurses do not go on duty at all on their "days off," which are given as nearly monthly as possible. Two weeks' holiday yearly are allowed to probationers, and three weeks to head nurses or sisters.

### Meals.

All meals except the night nurses' midnight tea, are served in the dining-room. The weekly supplies of butter, sugar, tea, &c., are kept in the dining-room, managed by the housekeeper, and no "allowances" are given into the nurses' own keeping. Nurses on day duty have a light breakfast at 6.30, and a meat breakfast at 9 to 9.30 a.m. Dinner is at 1 and 1.30 p.m., tea at 4 and 4 30, and supper at 9 p.m.

---

### SHEFFIELD ROYAL INFIRMARY (247 Beds).

### Terms of Training.

THE age considered desirable for probationers at the Sheffield Royal Infirmary is between 22 and 32 years. If accepted after two months' trial candidates enter upon a two years' engagement, a certificate being given at its satisfactory completion, and after passing examination. No premium is required from probationers, who are paid a small salary during the two years of their training—£6 the first and £9 the second year. Probationers who pass satisfactorily through this period have the option of remaining a third year as staff nurses, receiving £20 for the third year and a three years' certificate. At the completion of the third year nurses are eligible for promotion to the post of ward sister, such appointments being made solely on the ground of special fitness for a vacant position. Sisters' salaries range from £30 to £36 per annum. Laundry and indoor uniform are provided for the nursing staff. Lectures are given by the senior house surgeon and the matron on physiology, anatomy, nursing, and hygiene. Night duty is taken by first and second year nurses for three months at a time, usually at the end of the first year, and again in six or eight months' time. Third year nurses take night and day duty alternately in periods of two months. For promotion to the post of sister of wards, three years' training in an accredited general hospital is an essential qualification. All probationers are on the same footing, and alike eligible for promotion if they possess the requisite capacity and are fully trained.

### Hours On and Off Duty.

The probationers' average working day is ten hours, deducting off duty and meal times, and nine and six hours alternately on Sundays. Staff nurses on four days in the week are on duty for eleven hours, on two other days for six and nine hours, and on Sundays for six and nine hours alternately, they preferring an arrangement by which they have one half-day and one evening in the week, on other days being only sure of one hour instead of two off duty. The working hours of sisters average nine; they have a half day once a week, and an evening, with a half-day on alternate Sundays. All the staff have one clear day off duty once a month. Sisters have one month's annual leave, and the nurses three weeks and two days, the extra days being allowed for travelling. Night nurses are on duty from 9 p.m. to 8.30 a.m.

### Meals.

All meals are served in the dining-room, and there is no system of "allowances." As much food as may be required is served at each meal. The hours for day nurses' meals are: Breakfast, 6 30; luncheon, 9.30; dinner, 1 p.m.; tea, 4 30; and supper, 8.30. The sisters breakfast at 8.30; their lunch is served at 10 a.m.; dinner at 1.30; tea at 5; and supper at 9 p.m.

---

### Minor Appointments.

THE GUEST HOSPITAL, DUDLEY.—Miss A. Bartlett has been appointed Sister of the men's wards and of the theatre. She was trained at the Devon and Exeter Hospital and at the York Road Lying-in Hospital, Lambeth. Her nursing career comprised posts as sister and staff nurse at the Poplar Hospital, and night superintendent, Suffolk County Hospital.

ST. SAVIOUR'S INFIRMARY, EAST DULWICH.—Miss Agnes Gardiner, who trained at the Royal Infirmary, Liverpool, has been appointed Night Superintendent here. Miss Gardiner was for nearly two years charge nurse at the Carnarvonshire and Anglesey Infirmary, and for over two years ward sister at the Brentford Union Infirmary, Isleworth.

THE MILITARY INFECTIOUS HOSPITAL, ALDERSHOT.—The appointment of Nurse, recently vacant, has been given to Miss C. Seebohm, who was trained in the Manchester Workhouse Infirmary, and has been since at the North-Eastern Hospital (Metropolitan Asylums Board).

HOSPITAL OF ST. CROSS, RUGBY.—Miss Gwendda Evans has been appointed Charge Nurse here. She was trained at the Grimsby Hospital, and has been charge nurse at the North Staffordshire Infirmary, Stoke-on-Trent.

LEWISHAM INFIRMARY.—Miss Mary Winmill was appointed Sister here on December 19th. She has had four years' training at St. Bartholomew's Hospital, London.

BRENTFORD UNION INFIRMARY, ISLEWORTH, W.—Miss Kate Smith, who trained at the Leeds General Infirmary, was appointed Ward Sister at this infirmary on December 14th.

### Everybody's Opinion.

[Correspondence on all subjects is invited, but we cannot in any way be responsible for the opinions expressed by our correspondents. No communication can be entertained if the name and address of the correspondent is not given, as a guarantee of good faith but not necessarily for publication, or unless one side of the paper only is written on.]

### THE DIFFICULTY OF RETAINING DISTRICT NURSES.

"D. N." would like to fully corroborate all that "F. R. A." writes on the above. Does she know the following lines from Miller's "Secrets of a Beautiful Life"?

"A heart beat in our midst that vainly tried
Companionship of other hearts to gain;
A soul lived pure and sweet before our eyes
Whom our unsympathy caused cruel pain."

A district nurse would do well at the outset of her career as such to pause and remember that, in the majority of districts, a nurse is not taken up socially, no matter how well educated and refined she may be. If she cannot cheerfully carry, too, the burden of loneliness, she had better not begin district nursing. Only a real love for the suffering poor will ever retain a district nurse after she has served her time (the writer refers to "Queen's" nurses). The work is interesting and varied, but only she who lives the higher life, and as opportunity occurs strives to influence her patients (not by preaching, but by a happy, bright face and manners), will ever remain long a district nurse. Again, should the highly-trained district nurse after her term of service is up be out of work, she, in nine cases out of ten, stands no better chance of obtaining a post than one with less training. Unlike the district nurse in Holland, there is no pension to look forward to from headquarters—no inducement any way. The only pension she can hope for is from the R.N.P.F. if able to save; difficult at all times, but doubly so if out of employment.

SISTER LUCIE writes: I feel very grateful to "F. R. A." for ventilating some of the difficulties of district nurses. I have had several experiences in district nursing, and until six months ago (when I came to my present appointment) I have always received kindly consideration, and I have made many real friends who treated me as a gentlewoman and who did not regard me as a mere nursing machine. They helped me in the many little ways that make or mar the comfort of a nurse's life, and I fully appreciated their kindness. My present experience has been quite the reverse. Three or four of the ladies of the committee did call upon me, but it was too great a tax upon their memory to remember my name, and they talked about my cases and the sins of my patients until I felt I could hardly refrain from asking them to go. The nurse who preceded me was a woman of the old stamp of nurses. She mismanaged the work and was not always quite temperate. The district includes some of the dirtiest and lowest people, but now the medical men are helping me and the work is more satisfactory. It is the lack of a little sympathy and personal interest, the want of intercourse with people of our own class, the want of home life, that makes the life of town nurses so lonely and their work at times unbearable. A nurse is only a woman, and after a hard day's work amongst painful scenes, to return to dreary lodgings, and to spend her time off duty thinking of the home and friends she has left, does not brace her for the needs of the coming morrow. We do not want to be patronised by our social inferiors; we do not ask their hospitality; but there would be fewer changes amongst district nurses if intelligent kindly people would exert themselves a little, and would remember that it is not courteous to regard a nurse as having no idea beyond her work. Let her forget it and leave it and her uniform behind for a few hours, and she will resume her duties and do better work for the relaxation.

### THE PURITY OF MILK.

"G. R. B." writes: "Isola" will find the Lister-Gerber test both cheap and accurate. The apparatus consists of a revolving tin case fitted with springs to hold the test bottles. These have a scale on the neck. In them is placed 10 cc. of sulphuric acid with a specific gravity of not less than 1·825 or more than 1·828; add to this 1 cc. amyl alcohol and 11 cc. milk; cork up the bottle and shake gently till the contents are thoroughly mixed and no floating portions of curd are visible. Now place the bottles in the spring clips, screw on the cover, and revolve the machine for two or three minutes, when the bottles may be taken out and the result easily read, the fat being found in the neck of the bottle where the scale is. This test is especially useful for an institution, as abstraction of fat is the chief thing to guard against. Its simplicity makes it all the more valuable, as in very inexperienced hands one can hope for accurate results. It is, however, very essential that a fair sample should be taken. The whole of the milk should be thoroughly mixed by stirring, or, in the case of only a few gallons, by pouring it from one vessel to another. Ten minutes would be all the time required for a couple of tests, the accuracy of which is amply proved by the fact that most of the large dairy companies use it, and, having bought 3,000 imperial gallons daily, pay for it on the percentage of fat as shown by this test. I have every confidence in recommending it to any readers of THE HOSPITAL requiring a simple, quick, and reliable test. For the benefit of those not used to manipulating corrosives, I may add that it is useful to keep a bottle of ammonia handy with which to saturate any spots of acid which may find their way on to one's clothes. If this be used quickly enough it will prevent holes or loss of the original colour.

### ONE-SIDED ISOLATION.

"A. L. M." writes: May I be permitted to write a short article on isolation. I have not been trained in a fever hospital, but I have had a great deal of experience in nursing scarlet fever, diphtheria, and other infectious diseases. I have always been most particular both with regard to my patient, ward, room, and self, and have carried out all regulations laid down for nursing infectious diseases, also that one which is thought so absolutely necessary for the nurses, but not for the doctors, in not seeing any of one's friends. Doctors at the present time in practice are allowed to go from house to house after visiting an infectious case, and nothing is said because "he is the doctor." This no doubt cannot be helped, but, at the same time, why should medical men not take even more precaution than a nurse, who does not go from house to house? Why should they not, before entering a ward or bed-room, put on a large overall, which could be kept outside the carbolised sheet especially for them? If it is so essential that a nurse, who wears cotton dresses (clean every week), large aprons (clean every day), and when off duty changes into a clean dress, should not be allowed to speak to her own friends, why is it that the doctor can visit his patients in woollen clothing, into which germs from the patient or the patient's bedclothing are more likely to get? In case of scarlet fever or diphtheria, are not doctors, examining a patient's throat, just as likely to get the infectious part on themselves as the nurse? It will be said, no doubt, that they are careful, and on leaving a patient always wash their hands, but so is nurse. The doctors do not have their suits washed frequently, neither do they have new ones every day. The manner in which the disinfecting was done came under my notice whilst nursing in a country isolation hospital. The man who did it wore the same clothing as always, without any extra covering. If cloth carries infection so greatly, why, then, that man's clothes must have been full of germs handling so much scarlet fever and diphtheria clothing. To my knowledge he did six different lots of clothing which had been sent from town and district, and then he, above all others, and no one else was sent to do all the shopping. To me, isolation, at present, appears rather one-sided; the only one who is really kept at bay is the nurse. Doctors will, of course, say that infection might be in the nurse's system, and that is the reason she should not mix with her friends. But this might be the case with the doctor. There have been many doctors that have taken the infection from a patient. Again, if the clothing is so infectious, why is the man who does the disinfecting not just as likely as anyone else to take it and spread it? And yet he is a free man. I do not know what is the custom in London, but I am speaking of facts which I do know about in the country. It is certainly not the nurses who spread infection, but those who are allowed to nurse their own at home, who wear stuff dresses, and are far from being careful, both in themselves and in mixing with their friends. I can tell of an instance in which the mother and father went straight from an infectious house and travelled to bury a daughter who had caught the infection of diphtheria from one they had just buried at home. Besides that there were two from the same house that I was nursing in the hospital, and one child at home who developed it three weeks after. I fetched her from the home, and that very same day the mother had sent a girl of twelve straight from that infectious house to the dressmaker's, about two miles off. That is how infection is carried, and not by the careful nurse. A great deal more could be said on isolation.

THE INTERNATIONAL COUNCIL OF WOMEN.

R. E. TITHERINGTON writes: In THE HOSPITAL for December 17th, under the heading "A Word of Warning," you publish a letter from the secretary of the International Council of Women, in which a list is given of "well-known ladies" who "have been invited to take seats on the Professional Section Committee." There is reason to believe that several of the ladies there mentioned have declined the invitation, and the question of representation, therefore, is becoming a matter of serious importance to members of the nursing profession in England. It would be very desirable if information could be obtained from the secretary of the International Council as to the present membership of the "Professional Section Committee."

ROYAL NATIONAL PENSION FUND FOR NURSES.

"AN UNSATISFIED POLICYHOLDER" writes: May I through your columns ask why nurses who out of their hard-earned money have put their savings into the Royal National Pension Fund (which is by the public looked upon as a charity, not a company who has to pay dividends, &c.) if they die, say, within a year after their pension has become due, the whole of their hardly-earned capital should go? What, I ask you, is the good of such a Fund to poor nurses who have most of them relatives whom they would wish to help? Surely it ought to be possible that some of the money which they have reaped no benefit whatever from should go to their own relations. It strikes me as being a Fund likely to make nurses very selfish, and it makes them quite helpless in doing anything whatever for any poor relative they may have. Surely those who have given such large sums cannot really think that a nurse is justified in sinking her "all" with no chance of being able to leave anything at all to some poor and, perhaps, invalid relative at her death. Trusting you will pardon my encroaching upon your valuable space.

*** What the nurses subscribe for is an annuity, not a sum down, and it is quite clear that in calculating the amount of annuity which can be afforded both long and short lives have to be considered. It is, in fact, only because some annuitants die soon that others who live long can be paid the sums they in fact receive, and no such assurance association could maintain its position if it returned the money as "An Unsatisfied Policyholder" suggests. This lady, like a good many other persons, vulgarly speaking, wants to have her cake and eat it. She wishes to invest her money to produce a rate of interest very much higher than she could possibly obtain by any other safe means, and then if circumstances occur preventing her from drawing this high rate for a sufficiently long period, she wishes to have the alternative of receiving back (or rather of her representatives receiving) the whole, or part, of her capital. On the other hand, should the nurse live to a ripe old age, then the Fund is to make a loss. Such a course would be quite impracticable.—ED. *T. H.*

RURAL NURSES.

"A PROTESTER" writes: I have noted with great pleasure the fact that THE HOSPITAL has drawn attention to the grave dangers arising from a system such as that advocated by the "Bedfordshire Rural Nursing Association." This county should not, however, be credited with absolute originality as regards the institution of this "cheap manufactory of unqualified nurses." We are only feeble imitators of a system which was, I believe, first started by Lady Winchilsea, and which, if report speaks true, is fast spreading through counties where short-sighted philanthropy is combined with wealth. Four young village women have been trained for Bedfordshire. Two failed in their examination, and one of these two intends, I believe, to give up nursing. Those who are actually at work are, I hear, far from satisfied with the salary. It must be remembered that these girls do not live at home, and although uniforms are provided, still they have to find board and lodging out of 12s. a week. It will require a very high moral tone to refrain from taking bribes with so small a salary. One of these rural nurses was discussing this very matter with me the other day. She told me that she entered under the impression that the salary commenced at £30 per annum and rose to £50. Since then she had heard that she was only to receive 12s. a-week during three years. In her own home she has been regarded as a thrifty, hard-working girl, but she frankly told me, "I don't know how I'll ever manage on 12s. a week." Personally I do not believe that they are worth more than £30 per annum, but the system that can turn them out worth so little seems to me to speak for itself. The admiration I have for the fully-trained Jubilee nurse causes me to regret all the more deeply the fact that they have allowed these undertrained women to be affiliated to them. The average person knows no difference between a Jubilee nurse and one who is merely "affiliated." More than once I have had to explain to vicars' wives and people of that standing that a cottage nurse and a Jubilee nurse are not the same. I cannot understand why the cottage nurses, who must of necessity hold their L.O.S., cannot be satisfied with calling themselves midwives, without posing as trained nurses. For a time at least the fully-trained nurses must suffer, and as yet they have held their peace. The opinion of outsiders like myself may perhaps avail nothing, but it seems to us a thousand pities that these partially trained women should have received the *cachet* of so influential a body as the Queen's nurses.

### Warrington Workhouse.

RESIGNATION OF NURSES.

THE resignation of the superintendent nurse and of another nurse at the Warrington Workhouse came up for discussion at a recent meeting of the Board of Guardians. Some of the remarks made by one of the Guardians are instructive, showing, as they do, how unworkable is the present condition of affairs in country unions. Mr. Jolley, in reply to a question, explained that the new order of the Local Government Board had led to an increased demand for trained nurses, and that at Warrington "they could not offer decent accommodation, either for the sick poor, or for those paid to nurse them." Mr. Jolley added that "the superintendent nurse had tried to do her best, but she had been hampered in every way, and there had been constant sources of friction and trouble. It was inevitable that the master and matron should be brought into conflict occasionally with the head of the nursing staff. . . . He did not blame either the master and matron, or the superintendent nurse. Such friction as had arisen was inevitable." And the same tale will continue to be told so long as the Local Government Board elect to leave the superintendent nurse in her present impossible position. But it is satisfactory to know that the Warrington Guardians are pushing forward with their new hospital, and when that is completed no doubt the nursing difficulties will be considerably lessened.

### For Reading to the Sick.

LOVE, HEAVENBORN.

Love is of God.

WITH open love
The Lord of hearts would bless;
With age-long gladness, deep and sure,
With wealth of tenderness. —*MacDonald.*

From earth-born limits holding God-granted love in thrall!
From niggardly reserves, from offering less than all;
From the remorse that burns when life is past recall—
Shield Thou, O Perfect Love!

From littleness of aim, which dwarfs Thy bounteous gift;
From the harsh thoughts which cleave the swiftly-widening rift;
From hearts content with dust, when Thou would'st fain uplift—
Save Thou, O Perfect Love!

From meting forth Thy Love with scant and sordid measure;
From doubt lest He who gave may grudge His children's pleasure;
From fear lest Love Divine resent such earthly treasure—
Spare Thou, O Perfect Love!

Thou, Love—who for Love's sake did'st erst vouchsafe to die—
Through this our joy reveal the Face of the Most High;
Lead through each chast'ning fall, each hard-wrung victory,
On, on to Perfect Love!

*E. M. L. G.*

Love divine! all love excelling,
Joy of heaven! to earth come down,
Fix in us Thy humble dwelling,
All Thy faithful mercies crown!
Jesu, Thou art all compassion,
Pure, unbounded love Thou art;
Visit us with Thy salvation!
Enter every waiting heart. —*C. Wesley.*

**Reading.**

The nearer the soul approaches to the divine and eternal source of love, the more fully do the obligations of sacred human love reveal themselves, and the more keen is the self-reproach for the neglect even of the smallest of these.—*Catherine of Siena.*

Love is the greatest thing that God can give us, for Himself is love; and it is the greatest thing we can give to God, for it will also give ourselves and carry with it all that is ours. The Apostle calls it the band of perfection; it is the old, and it is the new, and it is the great commandment, and it is all the commandments; for it is the fulfilling of the law. It does the work of all other graces without any instrument but its own immediate virtue.—*Jeremy Taylor.*

We are to have love like that of Christ. In one sense this is impossible. "Measure the waters in the hollow of thine hand; mete out heaven with a span; comprehend the dust of the earth in a measure; weigh the mountains on scales and the hills in a balance." These are measurable things, but the love of Christ is measureless. There is nothing in His nature which is not infinite. The depths of God are depths of love, for God is love and Christ is God. Till the less can include the greater, and the human the divine, we may not fathom the depths or grasp the dimensions of Christ's love. To love like Paul, to love like John, would be a lofty aim, but who can love like Christ? Let us not mistake His meaning. He asks not that our love shall equal His, but resemble His; not that it should be of the same strength, but of the same kind.—*Stanford.*

### Notes and Queries.

**The contents of the Editor's Letter-box have now reached such unwieldy proportions that it has become necessary to establish a hard and fast rule regarding Answers to Correspondents. In future, all questions requiring replies will continue to be answered in this column without any fee. If an answer is required by letter, a fee of half-a-crown must be enclosed with the note containing the enquiry. We are always pleased to help our numerous correspondents to the fullest extent, and we can trust them to sympathise in the overwhelming amount of writing which makes the new rules a necessity.**

**Every communication must be accompanied by the writer's name and address, otherwise it will receive no attention.**

*Dispenser's Salary.*

(133) What salary could one ask in getting a post as dispenser after passing the Apothecaries' Hall (assistants) examination?—*L. S.*

From £50 to £80 non resident.

*Male Nurse.*

(134) Would you be kind enough to give me, if possible, some information as to qualifying myself for a male nurse? Circumstances (chiefly monetary) have prevented my becoming a medical man but have not extinguished my love of the work. I am 24 years of age, 5 ft. 10 in. in height, and have received a fairly good education. I have obtained the third year St. John Ambulance certificate, which includes invalid transport, and I have a very fair amount of medical knowledge and pharmacy. Unfortunately, I have had a great experience with sickness in my own family.—*F. G. F.*

The only hospital training available for male nurses in England is that given by the National Hospital for the Paralysed and Epileptic, Queen's Square, Bloomsbury, W.C. A fair amount of training can be obtained in lunatic asylums, through which a certificate from the Medico-Psychological Society can be gained (address the Registrar, 11, Chardos Street, W.) Have you thought of qualifying as a dispenser? It is possible to earn a livelihood as chemist's assistant and read for the examinations of the Society of Apothecaries of London for assistants, (address, Blackfriars, E.C.)

*The Rule of Infirmaries.*

(135) Can you tell me if there is a stated rule belonging to poor law infirmaries where a three years' training is given? (2) How many midwifery cases a probationer nurse is supposed to see during that time?—*Subscriber.*

If you mean is there a universal code of rules for all poor law infirmaries with regard to the training of the nurses for a three year's certificate, there is not. (2) Neither is instruction in maternity nursing or midwifery compulsory. Candidates for the L.O.S. certificate must have attended 25 cases.

*Guardians' Subsidy.*

(136) Would you be kind enough to tell me if the Local Government Board will sanction guardians giving a donation to a local district nursing association. I think they will, but I want to be quite sure, and would like to have good authority for saying so. Also, could you tell me the names of any institutions that are so helped, or suggest any way in which I could find them out?—*Ecnirp.*

The Local Government Board frequently sanctions grants made by the guardians to district nursing associations if the circumstances warrant it. For instance, at Gloucester the grant reached the large sum of £150 at the end of the year in which small pox was prevalent. We often notice small subscriptions to local associations. But if you want ever to become a superintendent nurse you must undergo three years of training, and this must be at a recognized training school. See below, "Preparatory Instruction."

*The Red Cross.*

(137) Could you kindly tell me the address of the Secretary of the National Aid Society, and if that is the same as the Red Cross Society?—*A. L.*

The official title of the Red Cross Society is the National Society for Aid to the Sick and Wounded in War. The address is 4, York Chambers, Adelphi, W.C.

*Preparatory Instruction.*

(138) 1. Would you kindly tell me what books on nursing I might get, and (2) if there are lectures in ambulance and bandaging I could attend preparatory to entering a general hospital as probationer. 3. Should also like to know if certificate from hospital with 100 beds would be recognized as fully qualified.—*Louie Slee.*

We are sending you a list of the publications of the Scientific Press, London, which may help you in your selection. 2. The St. John Ambulance Association arranges "first aid" lectures and demonstrations at various centres; address the Secretary, St. John's Gate, Clerkenwell. 3. It depends upon the constitution of the hospital. Ask the matron if the hospital of which you require the information is recognised by the Local Government Board as a training school for nurses.

*Essential Training.*

(139) 1. Will you kindly tell me what training is necessary to become a Queen's Jubilee nurse? 2. Can you also tell me if the Chester General and Sunderland General Infirmaries are recognized training schools?—*Probationer.*

1. The qualifications for a Queen's nurse are "not less than one year's training at an approved hospital or infirmary, with six months' approved training in district nursing." Apply Miss Peters, 1, St. Katherine's Royal Hospital, Regent's Park, N.W. 2. Yes

*Maternity Training.*

(140) I wish to be trained for maternity work. 1. Is it necessary that a pupil for the above should go through a course of training for sick nursing first? Will you recommend a hospital where the terms are moderate?—*E. E. K.*

1. No. Either the Clapham Maternity Hospital, Jeffrey's Road, Clapham (fees £25 4s., and extras), or the East-end Mother's Home (fees £15 15s ) would suit you.

### Travel Notes.

By Our Travelling Correspondent.

Rules in regard to Correspondence for this Section.—All questioners must use a pseudonym for publication, but the communication must also bear the writer's own name and address as well, which will be regarded as confidential. All such communications to be addressed "Travel Editor, 'Nursing Mirror,' 28, Southampton Street, Strand." No charge will be made for inserting and answering questions in the inquiry column, and all will be answered in rotation as space permits. If an answer by letter is required, a stamped and addressed envelope must be enclosed, together with 2s. 9d., which fee will be devoted to the objects of the "Hospital Convalescent Fund." Any inquiries reaching the office after Monday cannot be answered in "The Mirror" of the current week.

### III.—WHAT TO TAKE AND WHAT TO AVOID.

This is a thrilling question, and one on which one is always gaining new lights, achieving fresh successes, and, alas, experiencing fresh disasters. The articles taken differ considerably in quantity and character, according as to whether you mean to spend, say, six months in apartments or three in an hotel. First, then, let us consider the clients for hotels.

### Costume and Personal Outfit.

It is a fatal mistake to be insufficiently supplied with warm clothing; the inexperienced traveller imagines the sunny south to be also always the warm south; nothing can be a greater fallacy. I remember many a nipping day on the Riviera that even now makes me shudder to remember, because, in my want of knowledge, I had clothed myself for a fine spring day. It is often as cold as in England, only that the sun, being so constantly present, tempers the sharpness of the air, and one can always find a sheltered spot where one can enjoy friend Sol without being blown to pieces by a north-easter. Moreover, the absence of damp makes the cold exhilarating, and robs it of the danger that is its frequent accompaniment in more northern climes. To be comfortable and safe on the Riviera warm but light clothing is desirable. Nothing is so satisfactory to my mind for underclothing as woollen combinations, not too thick, supplemented by knickerbockers, tweed for the colder weather, and cotton or silk, not linen, for warmer days. About the body the same addition can be made by the adoption of a thin flannel or woven bodice for chilly days, which may be discarded as the weather settles into warmth; that is to say, if the outer dress be of adequate thickness. Never be tempted to cast aside the woollen combinations, however warm the weather, it is never safe to do so; in a few minutes, though there has been little or nothing to herald its approach, the formidable mistral may be upon you, enveloping you in clouds of dust, and searching out every unprotected corner with its cruel blast. Always wear woollen or silk stockings, never cotton or lisle thread. The keeping of the body and extremities warm is of much greater importance than we seem practically to allow.

### Suitable Evening Dress.

In these days of fascinating blouses it is very easy to attain a smart appearance in the evening without the criminal carelessness of allowing a delicate person to wear an open neck and short sleeves. By all means encourage such an one to take an interest in her dress, and to smarten herself up as she likes; it is most salutary to banish the thought of invalidism as much as possible, but it must be done judiciously. I received a cunning hint on this subject from an American nurse, which I will pass on for the benefit of others. It is this: Let all evening blouses of silk be made up with a thin flannel lining; they can be as smart as you like, but they will be safe. Muslin and chiffon can be treated in the same manner with a thin, cheap silk to hide the flannel. It is not considered good form to dress much for table d'hote, so that, with a change of blouses for ordinary occasions, one really smart dress for the theatre if the traveller is able to go there, should be enough.

### Tailor-made Dresses.

For every day wear nothing is so useful as these delightful garments, which, if well cut and chosen, meet all requirements of smartness. Let them be pure wool, and light, both as to colour and weight. One encounters a good deal of white dust along the Corniche which makes dark colours undesirable. I think four dresses should be quite enough for a winter abroad—two tailor-made, one thicker than the other, made with coat and waistcoat, is best, so as to admit of variations to an unlimited extent in vests and blouses; and two evening dresses. If means and luggage admit of it, white serge costumes are always extremely smart, and very suitable to the gay Promenade des Anglais. In buying parasols remember that white or light colours must be lined with a darker shade; the sun is so penetrating that a parasol of light tone throughout is very little protection from its rays, and the result is a most unbecoming frown and a constant headache. As to headgear, it is such a matter of individual taste that my courage fails me to attack it. I will refrain from all comment except an entreaty to reject anything striking, however pretty. It is vexing to hear in an undertone, "Oh! here comes the girl with the poppies," or, "I sat next to that woman with the bird-of-prey to-day at the band!" For outside wraps the ever-convenient golf cape is not to be bettered for cold weather and driving, and a light, warm, dust cloak, that will be shower-proof also, is essentially necessary. A lighter cape that can be carried over the arm and put on as evening approaches will be found to furnish all that is required. You will not encounter much mud or hard walking, therefore the clump soles of England are unnecessary. Two pairs of Oxford shoes and one pair of boots, with pretty indoor shoes, will last you six months, unless you are inveterate pedestrians. English boots and shoes are extremely dear on the Continent, and naturally so when you consider the customs.

### Washing is a seriously expensive item abroad if the locality is fashionable. It is never worth while to burden yourself with a full supply of underclothing. Washing is done with such extraordinary promptitude in foreign hotels that what you send to the laundress in the morning is returned in the evening; therefore, three changes will be found ample, and your trunks will not be crowded with unnecessary underclothing. Flannel or cashmere nightdresses should be worn; they are always more healthy, and in travelling one must remember the dangers of damp beds *en route*, which may be greatly obviated by wearing a long woollen nightdress, which can be made as pretty and dainty as one in cotton. Never trust your clothes without a thorough airing either by the fire or in the sun; they never come within the region of a fire in the laundresses' houses. Washing is generally well done with the exception of such articles as woollen vests and combinations, which owing to the manner of their treatment, being washed in cold water—in the river, indeed—and not dried rapidly are apt to develope a sudden tightness and adhesive narrowness very trying to the temper. If you have a maid with you it is worth while to have her wash these garments herself and dry them quickly before your own fire. If you find the charges too exorbitant and passing the patience of Job, complain to the head waiter, and he will usually get the bill, at any rate, slightly reduced.

### Household Needs.

Even in first-class hotels there are a few domestic comforts that you will do well to take with you. You are probably aware that soap is never supplied, and you will not like what you buy there; take a sufficiency with you. Provide yourself also with two or three packets of Hudson's soap in powder; apart from laundry operation it is invaluable for cleaning out teapots, milk jugs, &c. Chamber-maids have but limited ideas on the subject of scalding out pails, &c., and then a packet of Hudson is a treasure—in fact, like the flea of Punch's famous advertisement, "no person should be without one." A spirit kettle and lamp will be in your tea basket; add to its arrangements a folding saucepan, and on arrival at once purchase one of those metal jugs with basket-work handles, that stand heat so well. It is the native custom to fill them and stand them among the wood embers, and a very good plan it is, as hot water is thus constantly obtainable. Kettles, except very large, clumsy ones, are not used abroad. Take with you a small lamp for reading; nothing is more dismal than spending a long evening with the feeble illumination of hotel candles; true, there is generally electric light now, but it is very handy to have a lamp to place just where you want it, and where it can be shaded according to requirements, especially for night watching. Bath towels are not often met with, or, if present, are extremely small, so that it is well to take them with you.

### Needs in Apartments.

If you purpose living in rooms of your own, your requirements as to domestic necessities will be greater. Anything more meagre than the popular idea of the plenishing required in French furnished apartments it is impossible to conceive. Not in the matter of linen though. You need take nothing of that sort, with the exception of bath towels and tea and glass cloths. Kitchen cloths generally are called "torchons," and are limited in number as a rule. Small basons, small plates and jugs, and all those little conveniences so indispensable to an invalid are conspicuous by their absence. Another great addition to your comfort involving scarcely any added weight to your luggage, would be a couple of cheap Japanese trays. Trays are seldom found in furnished apartments, and if present are battered iron atrocities, large and cumbrous, and that give forth loud reports complainingly when lifted. Do not forget to take tea whether, residing in hotels or apartments. It is both dear and bad abroad. You are at liberty to take one pound with you if you declare it at the Customs as for your "propre service," and if two of you do this you will have enough, or nearly so, for your stay. Take a tin for holding it, and another for biscuits and the delicious little foreign cakes that are such a comfort for afternoon tea. This reminds me that afternoon teacloths are a decided want. Even in apartments do not trouble to carry teacups with you; they are cumbrous to pack, and can be bought very cheaply.

### Exchange of Money.

Change your money at Charing Cross before you start, and not on the boat. See that you do not get Belgian coins in France nor silver French coins previous to 1863. Twenty-five francs and a few centimes go to an English sovereign, and one feels, therefore, richer, because, roughly speaking, a franc is used pretty much as we use a shilling, and where in England we should give sixpence, in France we give half a franc. In Italy the rate of exchange always gives one a pleasing feeling of wealth because one usually obtains at least 27 lire for a sovereign, and in Spain the difference is so great as to encourage the agreeable sensation of one's purse being like the widow's cruse of oil. In this case, though, it is all important to bring back as little money as possible as the loss will be proportionately great in crossing the frontier again. The safest and simplest way to manage your money abroad is by circular notes or letters of credit. It obviates the danger and anxiety attendant upon carrying a large sum of money with you. The tourist agency through which you get your tickets will arrange this for you. You deposit the money with them in London, and take the circular notes with you to be signed and cashed whenever and wherever needed. By this arrangement it is unnecessary to take more money than enough for your journey with you, allowing a margin of a couple of pounds for emergencies. If you want money in a hurry, and you have drawn all you deposited, these confiding agents will send you more by telegram; at least, that has been my experience.

### Tips in Hotels.

This is a subject often extremely puzzling to travellers. Either they give so much in excess as to engender the idea that they are millionaires, or so little that they are regarded as models of meanness. It is a fair rule on which to go, subject to modifications, to give the head waiter at the rate of half a franc a day, which would be 3 fr. 50 a week, and the same to your chambermaid. If you stayed four months in an hotel give them £2 each, which would be 9 fr. 50 less than the half-franc daily; on the other hand, if you stayed three days in an hotel 1 fr. 50 is hardly enough; I should give 2 fr. 50 each. *Pourboires*, which mean a tip to the driver of your carriage, range from 20 centimes to 1 fr. or 1 fr. 50, according to the length of the drive; if you have a carriage the entire day the latter sum is amply sufficient. It is rather difficult to give a general rule as to tips for the hotel concièrge, because it all depends upon what he does for you; some are the embodiment of usefulness and courtesy, and do everything for you, from buttoning your boots to fighting exorbitant shopkeepers on your behalf, whilst others confine themselves to looking stolidly magnificent, and exercising a masterly inactivity. If he has been really useful and obliging I should give him a sovereign at the end of the season. I cannot impress on you too strongly the good policy of being on the best terms with all the servants of your hotel. It is very easily done; a pleasant look or gracious word of thanks goes far with them, and so much of your comfort will depend on their attitude, that it is wise, as well as kind, not to lose sight of their ability to make or mar much of your comfort.

